# {μ-6,6′-Dimeth­oxy-2,2′-[propane-1,3-diylbis(nitrilo­methanylyl­idene)]di­phenolato}dimethano­ltrinitrato­samarium(III)zinc(II) methanol disolvate

**DOI:** 10.1107/S1600536811007641

**Published:** 2011-04-07

**Authors:** Fei Liu, Fang Zhang

**Affiliations:** a The College of Chemical Engineering & Materials, Eastern Liaoning University, No. 325 Wenhua Road, Yuanbao District, Dandong City, Liaoning Province 118003, People’s Republic of China

## Abstract

In the title complex, [SmZn(C_19_H_20_N_2_O_4_)(NO_3_)_3_(CH_3_OH)_2_]·2CH_3_OH, the Zn^II^ ion is six-coordinated by two O atoms and two N atoms of the deprotonated Schiff base ligand and by two O atoms from methanol mol­ecules, forming a slightly distorted octa­hedral geometry. The Sm^III^ ion is coordinated by six O atoms from three chelating nitrate ligands and four O atoms from the Schiff base ligand, forming a distorted bicapped square-anti­prismatic environment. In the crystal, inter­molecular O—H⋯O hydrogen bonds connect the complex mol­ecules and the two methanol solvent mol­ecules, forming (10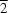) sheets.

## Related literature

For the isotypic Pr^III^/Ni^II^ complex, see: Liu & Zhang (2008 ▶) and for the isotypic Sm^III^/Ni^II^ complex, see: Liu (2009 ▶). For a related Sm^III^/Cu^II^ complex, see: Wang *et al.* (2008 ▶).
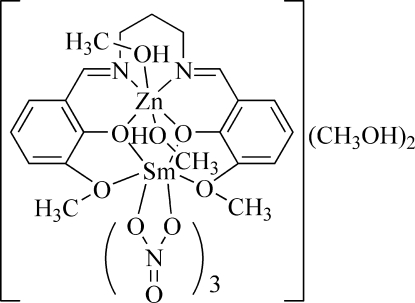


## Experimental

### 

#### Crystal data

[SmZn(C_19_H_20_N_2_O_4_)(NO_3_)_3_(CH_4_O)_2_]·2CH_4_O
*M* *_r_* = 870.29
Monoclinic, 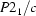

*a* = 13.1050 (8) Å
*b* = 11.1190 (7) Å
*c* = 22.3240 (13) Å
β = 90.999 (1)°
*V* = 3252.4 (3) Å^3^
*Z* = 4
Mo *K*α radiation
μ = 2.61 mm^−1^
*T* = 296 K
0.32 × 0.26 × 0.24 mm

#### Data collection

Rigaku R-AXIS RAPID CCD diffractometer
Absorption correction: multi-scan (*ABSCOR*; Higashi, 1995 ▶) *T*_min_ = 0.489, *T*_max_ = 0.573
23458 measured reflections
5833 independent reflections
4835 reflections with *I* > 2σ(*I*)
*R*_int_ = 0.073

#### Refinement

*R*[*F* ^2^ > 2σ(*F* ^2^)] = 0.070
*wR*(*F* ^2^) = 0.133
*S* = 1.46
5833 reflections
433 parameters
28 restraints
H-atom parameters constrained
Δρ_max_ = 0.92 e Å^−3^
Δρ_min_ = −1.14 e Å^−3^

### 

Data collection: *PROCESS-AUTO* (Rigaku, 2006 ▶); cell refinement: *PROCESS-AUTO*; data reduction: *CrystalStructure* (Rigaku, 2007 ▶); program(s) used to solve structure: *SHELXS97* (Sheldrick, 2008 ▶); program(s) used to refine structure: *SHELXL97* (Sheldrick, 2008 ▶); molecular graphics: *ORTEP-3 for Windows* (Farrugia, 1997 ▶); software used to prepare material for publication: *WinGX* (Farrugia, 1999 ▶).

## Supplementary Material

Crystal structure: contains datablocks global, I. DOI: 10.1107/S1600536811007641/zs2088sup1.cif

Structure factors: contains datablocks I. DOI: 10.1107/S1600536811007641/zs2088Isup2.hkl

Additional supplementary materials:  crystallographic information; 3D view; checkCIF report

## Figures and Tables

**Table 1:** Hydrogen-bond geometry (Å, °)

*D*—H⋯*A*	*D*—H	H⋯*A*	*D*⋯*A*	*D*—H⋯*A*
O5—H501⋯O8^i^	0.82	2.38	3.107 (11)	148
O6—H601⋯O17^ii^	0.82	1.99	2.654 (10)	138
O17—H17*A*⋯O16	0.82	1.88	2.688 (13)	167

Symmetry codes: (i) 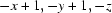; (ii) 
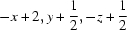
.

**Fig. 1.**
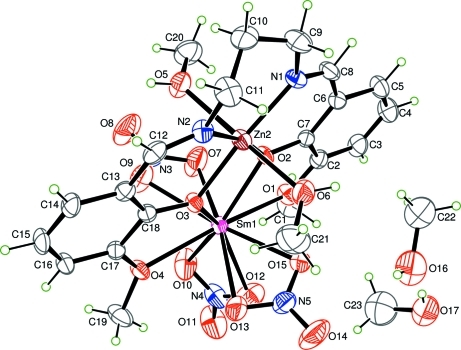
The molecular structure of (I), showing atom numbering scheme and probability displacement ellipsoids drawn at the 40% level. The dashed line indicates a hydrogen bond.

## supplementary crystallographic information

### Comment

The title complex, (I), formed with the Schiff base *N*,*N*'-bis(2-hydroxy-3-methoxybenzylidene)-1,3-diaminopropane is dinuclear with the Sm^III^ and Zn^II^ atoms linked through two phenolate O atoms from the ligand, and is the same as the bonding in the isostructural Pr^III^/Ni^II^ complex (Liu & Zhang, 2008) and the Sm^III^/Ni^II^ complex (Liu, 2009) with the same ligand as well as a related Sm^III^/Cu^II^ complex (Wang *et al.*, 2008).

In (I) (Fig. 1), the Sm^III^ ion is ten-coordinate, comprising four O atoms from the deprotonated Schiff base ligand and six O atoms from three chelating nitrate ions, forming a distorted bicapped square-antiprismatic environment. The Zn ^II^ ion has a slightly distorted six-coordinate stereochemistry comprising two N atoms and two O atoms from the Schiff base ligand and two methanol O atoms. There are two solvent methanol molecules for each complex molecule and in the crystal structure, intermolecular O—H···O hydrogen bonds (Table 1) connect these molecules to the complex molecules to form sheet structures extending along (1 0 -2).

### Experimental

The title complex was obtained by the treatment of zinc(II) acetate dihydrate (0.0548 g, 0.25 mmol) with the Schiff base [*N*,*N*'-bis(2-hydroxy-3-methoxybenzylidene)-1,3-diaminopropane)] (0.0855 g, 0.25 mmol) in methanol (25 ml) at room temperature. The mixture was refluxed for 3 h after the addition of samarium(III) nitrate hexahydrate (0.1111 g, 0.25 mmol). The reaction mixture was cooled and filtered. Diethyl ether was allowed to diffuse slowly into the solution of the filtrate giving colorless single crystals after several days.

### Refinement

H atoms bound to C atoms were placed in calculated positions and treated as riding on their parent atoms, with C—H = 0.93 Å (aromatic C), C—H = 0.97 Å (methylene C), C—H = 0.98 Å (methine C), and with *U*_iso_(H) = 1.2*U*_eq_(C) or C—H = 0.96 Å (methyl C) and with *U*_iso_(H) = 1.5*U*_eq_(C).

### Crystal data

**Table:**

[SmZn(C_19_H_20_N_2_O_4_)(NO_3_)_3_(CH_4_O)_2_]·2CH_4_O	*F*(000) = 1748
*M**_r_* = 870.29	*D*_x_ = 1.777 Mg m^−^^3^
Monoclinic, *P*2_1_/*c*	Mo *K*α radiation, λ = 0.71073 Å
Hall symbol: -P 2ybc	Cell parameters from 26435 reflections
*a* = 13.1050 (8) Å	θ = 3.0–28.1°
*b* = 11.1190 (7) Å	µ = 2.61 mm^−^^1^
*c* = 22.3240 (13) Å	*T* = 296 K
β = 90.999 (1)°	Block, colorless
*V* = 3252.4 (3) Å^3^	0.32 × 0.26 × 0.24 mm
*Z* = 4	

### Data collection

**Table:**

Rigaku R-AXIS RAPID CCD diffractometer	5833 independent reflections
Radiation source: rolling anode	4835 reflections with *I* > 2σ(*I*)
graphite	*R*_int_ = 0.073
Detector resolution: 10.00 pixels mm^-1^	θ_max_ = 25.2°, θ_min_ = 3.0°
ω scans	*h* = −14→15
Absorption correction: multi-scan (*ABSCOR*; Higashi, 1995)	*k* = −13→12
*T*_min_ = 0.489, *T*_max_ = 0.573	*l* = −26→26
23458 measured reflections	

### Refinement

**Table:**

Refinement on *F*^2^	Primary atom site location: structure-invariant direct methods
Least-squares matrix: full	Secondary atom site location: difference Fourier map
*R*[*F*^2^ > 2σ(*F*^2^)] = 0.070	Hydrogen site location: inferred from neighbouring sites
*wR*(*F*^2^) = 0.133	H-atom parameters constrained
*S* = 1.46	*w* = 1/[σ^2^(*F*_o_^2^) + (0.*P*)^2^ + 11.1281*P*] where *P* = (*F*_o_^2^ + 2*F*_c_^2^)/3
5833 reflections	(Δ/σ)_max_ = 0.001
433 parameters	Δρ_max_ = 0.92 e Å^−^^3^
28 restraints	Δρ_min_ = −1.14 e Å^−^^3^

### Special details

**Table:**

Geometry. All e.s.d.'s (except the e.s.d. in the dihedral angle between two l.s. planes) are estimated using the full covariance matrix. The cell e.s.d.'s are taken into account individually in the estimation of e.s.d.'s in distances, angles and torsion angles; correlations between e.s.d.'s in cell parameters are only used when they are defined by crystal symmetry. An approximate (isotropic) treatment of cell e.s.d.'s is used for estimating e.s.d.'s involving l.s. planes.
Refinement. Refinement of *F*^2^ against ALL reflections. The weighted *R*-factor *wR* and goodness of fit *S* are based on *F*^2^, conventional *R*-factors *R* are based on *F*, with *F* set to zero for negative *F*^2^. The threshold expression of *F*^2^ > σ(*F*^2^) is used only for calculating *R*-factors(gt) *etc*. and is not relevant to the choice of reflections for refinement. *R*-factors based on *F*^2^ are statistically about twice as large as those based on *F*, and *R*- factors based on ALL data will be even larger.

### Fractional atomic coordinates and isotropic or equivalent isotropic displacement parameters (Å^2^)

**Table:**

	*x*	*y*	*z*	*U*_iso_*/*U*_eq_	
Sm1	0.71666 (3)	0.20224 (3)	0.077898 (18)	0.02935 (14)	
Zn2	0.67371 (7)	0.48266 (8)	0.14718 (4)	0.0333 (2)	
O4	0.5366 (4)	0.1137 (5)	0.0902 (3)	0.0377 (13)	
O1	0.9006 (4)	0.2424 (5)	0.0458 (3)	0.0399 (14)	
O3	0.6021 (4)	0.3198 (4)	0.1324 (2)	0.0295 (12)	
O2	0.7805 (4)	0.3970 (4)	0.0965 (2)	0.0296 (12)	
C17	0.4629 (6)	0.1920 (7)	0.1110 (3)	0.0314 (17)	
N1	0.7663 (5)	0.6321 (5)	0.1551 (3)	0.0354 (16)	
O5	0.5971 (4)	0.5586 (6)	0.0634 (2)	0.0510 (16)	
H501	0.5345	0.5643	0.0629	0.076*	
C18	0.5021 (6)	0.3023 (7)	0.1337 (3)	0.0296 (16)	
O15	0.8284 (5)	0.1789 (5)	0.1690 (3)	0.0494 (16)	
N2	0.5487 (5)	0.5433 (6)	0.1907 (3)	0.0342 (15)	
C10	0.6117 (7)	0.7520 (8)	0.1875 (4)	0.050 (2)	
H10A	0.5942	0.8303	0.2035	0.060*	
H10B	0.5852	0.7483	0.1467	0.060*	
O10	0.7134 (6)	0.0288 (7)	0.0057 (4)	0.074 (2)	
C2	0.9462 (6)	0.3500 (7)	0.0634 (4)	0.0354 (19)	
C13	0.4309 (6)	0.3833 (7)	0.1582 (3)	0.0317 (17)	
N3	0.6467 (7)	0.2925 (7)	−0.0431 (4)	0.053 (2)	
N5	0.7745 (6)	0.1072 (7)	0.1988 (4)	0.0495 (19)	
C7	0.8776 (6)	0.4305 (7)	0.0910 (3)	0.0307 (17)	
O12	0.8114 (7)	−0.0017 (6)	0.0800 (4)	0.079 (2)	
O9	0.5881 (5)	0.2710 (7)	−0.0004 (3)	0.065 (2)	
O14	0.8046 (6)	0.0691 (8)	0.2471 (3)	0.092 (3)	
O7	0.7385 (6)	0.2794 (8)	−0.0320 (4)	0.081 (2)	
O6	0.7404 (5)	0.4206 (6)	0.2342 (3)	0.0532 (17)	
H601	0.8002	0.4375	0.2417	0.080*	
C5	1.0224 (7)	0.5656 (9)	0.1023 (4)	0.048 (2)	
H5	1.0494	0.6385	0.1156	0.058*	
C6	0.9172 (6)	0.5416 (7)	0.1100 (4)	0.0352 (19)	
C4	1.0844 (7)	0.4845 (9)	0.0762 (5)	0.054 (3)	
H4	1.1532	0.5023	0.0717	0.065*	
C3	1.0465 (7)	0.3753 (9)	0.0561 (4)	0.046 (2)	
H3	1.0892	0.3201	0.0379	0.055*	
O11	0.7952 (6)	−0.1404 (6)	0.0138 (4)	0.091 (3)	
C11	0.5599 (8)	0.6572 (8)	0.2237 (4)	0.052 (2)	
H11A	0.5992	0.6431	0.2602	0.063*	
H11B	0.4929	0.6858	0.2349	0.063*	
C1	0.9559 (7)	0.1707 (8)	0.0029 (5)	0.057 (3)	
H1A	0.9819	0.2220	−0.0278	0.086*	
H1B	0.9107	0.1121	−0.0148	0.086*	
H1C	1.0116	0.1304	0.0229	0.086*	
C16	0.3594 (6)	0.1679 (7)	0.1095 (4)	0.038 (2)	
H16	0.3354	0.0958	0.0936	0.046*	
C12	0.4594 (7)	0.4961 (7)	0.1871 (4)	0.037 (2)	
H12	0.4070	0.5389	0.2049	0.045*	
C14	0.3268 (6)	0.3562 (8)	0.1560 (4)	0.040 (2)	
H14	0.2804	0.4108	0.1715	0.048*	
O8	0.6132 (7)	0.3274 (8)	−0.0915 (3)	0.096 (3)	
C15	0.2921 (6)	0.2508 (8)	0.1317 (4)	0.045 (2)	
H15	0.2223	0.2351	0.1300	0.054*	
C8	0.8570 (7)	0.6358 (7)	0.1382 (4)	0.040 (2)	
H8	0.8906	0.7086	0.1445	0.048*	
C9	0.7269 (8)	0.7413 (8)	0.1862 (5)	0.053 (3)	
H9A	0.7540	0.8120	0.1667	0.064*	
H9B	0.7530	0.7410	0.2272	0.064*	
O13	0.6889 (5)	0.0797 (6)	0.1765 (3)	0.064 (2)	
C20	0.6512 (8)	0.5994 (9)	0.0100 (3)	0.064 (3)	
H20A	0.6688	0.6828	0.0144	0.097*	
H20B	0.6079	0.5893	−0.0248	0.097*	
H20C	0.7122	0.5527	0.0054	0.097*	
N4	0.7733 (6)	−0.0421 (7)	0.0331 (4)	0.055 (2)	
C21	0.6852 (9)	0.3553 (10)	0.2808 (4)	0.079 (4)	
H21A	0.6235	0.3219	0.2639	0.119*	
H21B	0.6685	0.4097	0.3126	0.119*	
H21C	0.7274	0.2917	0.2964	0.119*	
C19	0.5092 (7)	−0.0105 (7)	0.0849 (5)	0.059 (3)	
H19A	0.4769	−0.0362	0.1210	0.089*	
H19B	0.5694	−0.0576	0.0787	0.089*	
H19C	0.4628	−0.0208	0.0516	0.089*	
O17	1.1227 (7)	0.0443 (8)	0.2022 (4)	0.091 (3)	
H17A	1.0920	0.1050	0.2124	0.136*	
C23	1.0781 (12)	−0.0015 (13)	0.1456 (5)	0.118 (5)	
H23A	1.0169	−0.0455	0.1538	0.178*	
H23B	1.1262	−0.0536	0.1266	0.178*	
H23C	1.0622	0.0648	0.1195	0.178*	
C22	1.0335 (10)	0.3366 (9)	0.2225 (6)	0.091 (4)	
H22A	1.0513	0.3268	0.1812	0.136*	
H22B	1.0908	0.3689	0.2445	0.136*	
H22C	0.9767	0.3907	0.2252	0.136*	
O16	1.0056 (8)	0.2192 (8)	0.2477 (5)	0.105 (3)	
H16A	0.9525	0.2259	0.2664	0.157*	

### Atomic displacement parameters (Å^2^)

**Table:**

	*U*^11^	*U*^22^	*U*^33^	*U*^12^	*U*^13^	*U*^23^
Sm1	0.0255 (2)	0.0275 (2)	0.0350 (2)	0.00215 (19)	−0.00014 (15)	−0.00476 (18)
Zn2	0.0302 (5)	0.0289 (5)	0.0409 (6)	−0.0001 (4)	0.0052 (4)	−0.0079 (4)
O4	0.030 (3)	0.028 (3)	0.055 (4)	−0.005 (2)	0.004 (3)	−0.005 (3)
O1	0.031 (3)	0.039 (3)	0.051 (4)	0.000 (3)	0.009 (3)	−0.008 (3)
O3	0.020 (3)	0.027 (3)	0.041 (3)	0.002 (2)	0.004 (2)	−0.006 (2)
O2	0.021 (3)	0.027 (3)	0.041 (3)	−0.003 (2)	0.008 (2)	−0.006 (2)
C17	0.030 (4)	0.035 (4)	0.029 (4)	−0.003 (4)	0.000 (3)	0.004 (3)
N1	0.041 (4)	0.026 (4)	0.039 (4)	−0.004 (3)	0.001 (3)	−0.004 (3)
O5	0.038 (4)	0.070 (4)	0.045 (4)	0.006 (3)	−0.001 (3)	0.012 (3)
C18	0.031 (4)	0.033 (4)	0.024 (4)	−0.003 (4)	0.000 (3)	0.004 (3)
O15	0.051 (4)	0.048 (4)	0.049 (4)	−0.009 (3)	−0.011 (3)	0.008 (3)
N2	0.032 (4)	0.035 (4)	0.035 (4)	0.005 (3)	0.003 (3)	−0.009 (3)
C10	0.061 (7)	0.032 (4)	0.057 (6)	0.014 (5)	−0.001 (5)	−0.015 (4)
O10	0.058 (5)	0.066 (5)	0.097 (6)	0.021 (4)	−0.019 (4)	−0.043 (4)
C2	0.031 (5)	0.040 (5)	0.035 (5)	0.002 (4)	0.001 (4)	0.004 (4)
C13	0.032 (4)	0.036 (4)	0.028 (4)	0.003 (4)	0.004 (3)	0.004 (3)
N3	0.055 (5)	0.055 (5)	0.049 (5)	0.007 (4)	−0.012 (4)	0.007 (4)
N5	0.045 (5)	0.054 (5)	0.050 (5)	0.008 (4)	0.001 (4)	0.012 (4)
C7	0.026 (4)	0.036 (4)	0.030 (4)	−0.004 (3)	−0.001 (3)	0.009 (3)
O12	0.109 (7)	0.059 (5)	0.068 (5)	0.026 (4)	−0.015 (5)	−0.012 (4)
O9	0.050 (4)	0.093 (6)	0.052 (4)	0.017 (4)	−0.001 (4)	0.010 (4)
O14	0.091 (6)	0.124 (7)	0.061 (5)	0.016 (5)	−0.012 (4)	0.045 (5)
O7	0.044 (4)	0.125 (7)	0.074 (5)	0.011 (5)	−0.001 (4)	0.033 (5)
O6	0.048 (4)	0.064 (4)	0.047 (4)	−0.007 (3)	−0.007 (3)	0.010 (3)
C5	0.036 (5)	0.053 (6)	0.057 (6)	−0.016 (4)	−0.003 (4)	0.004 (5)
C6	0.036 (5)	0.035 (4)	0.035 (4)	−0.006 (4)	−0.005 (4)	0.006 (3)
C4	0.024 (5)	0.068 (7)	0.070 (7)	−0.008 (5)	0.008 (5)	0.006 (5)
C3	0.029 (5)	0.057 (6)	0.052 (6)	0.005 (4)	0.006 (4)	0.004 (4)
O11	0.065 (5)	0.054 (5)	0.155 (8)	0.016 (4)	0.002 (5)	−0.056 (5)
C11	0.052 (6)	0.051 (6)	0.054 (6)	0.004 (5)	0.009 (5)	−0.022 (5)
C1	0.050 (6)	0.053 (6)	0.069 (7)	0.009 (5)	0.024 (5)	−0.012 (5)
C16	0.030 (5)	0.042 (5)	0.043 (5)	−0.009 (4)	−0.003 (4)	0.002 (4)
C12	0.039 (5)	0.041 (5)	0.032 (4)	0.016 (4)	0.003 (4)	0.000 (4)
C14	0.022 (4)	0.052 (5)	0.046 (5)	0.005 (4)	0.005 (4)	0.005 (4)
O8	0.098 (7)	0.134 (8)	0.055 (5)	0.017 (6)	−0.023 (5)	0.034 (5)
C15	0.019 (4)	0.060 (6)	0.056 (6)	−0.004 (4)	0.000 (4)	0.003 (5)
C8	0.045 (6)	0.033 (5)	0.041 (5)	−0.017 (4)	−0.003 (4)	0.003 (4)
C9	0.065 (7)	0.032 (5)	0.063 (6)	−0.006 (5)	0.003 (5)	−0.013 (4)
O13	0.041 (4)	0.075 (5)	0.076 (5)	−0.003 (4)	−0.009 (4)	0.024 (4)
C20	0.072 (8)	0.072 (7)	0.049 (6)	0.003 (6)	0.005 (5)	0.020 (5)
N4	0.039 (5)	0.042 (5)	0.082 (7)	−0.002 (4)	0.015 (5)	−0.020 (4)
C21	0.092 (10)	0.082 (8)	0.064 (8)	−0.018 (7)	0.011 (7)	0.012 (6)
C19	0.046 (6)	0.029 (5)	0.103 (9)	−0.006 (4)	0.014 (6)	−0.006 (5)
O17	0.089 (4)	0.094 (4)	0.089 (4)	0.003 (4)	−0.019 (4)	0.003 (4)
C23	0.118 (7)	0.122 (7)	0.114 (7)	−0.002 (5)	−0.004 (5)	−0.001 (5)
C22	0.089 (6)	0.094 (5)	0.090 (6)	0.006 (4)	−0.004 (4)	0.005 (4)
O16	0.095 (5)	0.109 (5)	0.110 (5)	0.000 (4)	−0.014 (4)	0.001 (4)

### Geometric parameters (Å, °)

**Table:**

Sm1—O3	2.346 (5)	N5—O13	1.256 (9)
Sm1—O2	2.356 (5)	C7—C6	1.402 (10)
Sm1—O15	2.499 (6)	O12—N4	1.237 (10)
Sm1—O10	2.512 (6)	O6—C21	1.469 (12)
Sm1—O9	2.525 (6)	O6—H601	0.8208
Sm1—O1	2.565 (5)	C5—C4	1.353 (13)
Sm1—O4	2.576 (5)	C5—C6	1.418 (11)
Sm1—O12	2.586 (7)	C5—H5	0.9300
Sm1—O7	2.620 (7)	C6—C8	1.461 (12)
Sm1—O13	2.620 (7)	C4—C3	1.383 (13)
Sm1—N5	2.984 (8)	C4—H4	0.9300
Sm1—N4	2.993 (8)	C3—H3	0.9300
Zn2—N2	2.035 (6)	O11—N4	1.211 (9)
Zn2—O2	2.050 (5)	C11—H11A	0.9700
Zn2—N1	2.063 (6)	C11—H11B	0.9700
Zn2—O3	2.064 (5)	C1—H1A	0.9600
Zn2—O6	2.225 (6)	C1—H1B	0.9600
Zn2—O5	2.270 (6)	C1—H1C	0.9600
O4—C17	1.386 (9)	C16—C15	1.374 (12)
O4—C19	1.431 (9)	C16—H16	0.9300
O1—C2	1.391 (9)	C12—H12	0.9300
O1—C1	1.450 (10)	C14—C15	1.367 (12)
O3—C18	1.326 (8)	C14—H14	0.9300
O2—C7	1.333 (8)	C15—H15	0.9300
C17—C16	1.383 (11)	C8—H8	0.9300
C17—C18	1.419 (10)	C9—H9A	0.9700
N1—C8	1.255 (10)	C9—H9B	0.9700
N1—C9	1.495 (10)	C20—H20A	0.9600
O5—C20	1.469 (10)	C20—H20B	0.9600
O5—H501	0.8228	C20—H20C	0.9600
C18—C13	1.415 (10)	C21—H21A	0.9600
O15—N5	1.263 (9)	C21—H21B	0.9600
N2—C12	1.284 (10)	C21—H21C	0.9600
N2—C11	1.471 (10)	C19—H19A	0.9600
C10—C11	1.498 (13)	C19—H19B	0.9600
C10—C9	1.516 (13)	C19—H19C	0.9600
C10—H10A	0.9700	O17—C23	1.474 (15)
C10—H10B	0.9700	O17—H17A	0.8200
O10—N4	1.262 (10)	C23—H23A	0.9600
C2—C3	1.358 (11)	C23—H23B	0.9600
C2—C7	1.417 (11)	C23—H23C	0.9600
C13—C14	1.396 (11)	C22—O16	1.470 (14)
C13—C12	1.456 (11)	C22—H22A	0.9600
N3—O8	1.222 (10)	C22—H22B	0.9600
N3—O7	1.233 (10)	C22—H22C	0.9600
N3—O9	1.257 (10)	O16—H16A	0.8200
N5—O14	1.218 (9)		
			
O3—Sm1—O2	67.98 (17)	C11—C10—C9	114.5 (8)
O3—Sm1—O15	90.4 (2)	C11—C10—H10A	108.6
O2—Sm1—O15	75.57 (18)	C9—C10—H10A	108.6
O3—Sm1—O10	139.1 (2)	C11—C10—H10B	108.6
O2—Sm1—O10	145.1 (2)	C9—C10—H10B	108.6
O15—Sm1—O10	116.5 (2)	H10A—C10—H10B	107.6
O3—Sm1—O9	76.4 (2)	N4—O10—Sm1	99.5 (5)
O2—Sm1—O9	94.2 (2)	C3—C2—O1	123.9 (8)
O15—Sm1—O9	165.8 (2)	C3—C2—C7	122.9 (8)
O10—Sm1—O9	77.6 (2)	O1—C2—C7	113.2 (7)
O3—Sm1—O1	131.33 (16)	C14—C13—C18	119.9 (7)
O2—Sm1—O1	63.68 (17)	C14—C13—C12	116.4 (7)
O15—Sm1—O1	72.8 (2)	C18—C13—C12	123.7 (7)
O10—Sm1—O1	87.7 (2)	O8—N3—O7	123.3 (9)
O9—Sm1—O1	111.9 (2)	O8—N3—O9	121.1 (9)
O3—Sm1—O4	64.04 (16)	O7—N3—O9	115.6 (8)
O2—Sm1—O4	130.98 (16)	O14—N5—O13	122.7 (9)
O15—Sm1—O4	113.5 (2)	O14—N5—O15	120.6 (9)
O10—Sm1—O4	76.7 (2)	O13—N5—O15	116.7 (7)
O9—Sm1—O4	65.7 (2)	O14—N5—Sm1	175.8 (7)
O1—Sm1—O4	164.38 (17)	O13—N5—Sm1	61.1 (4)
O3—Sm1—O12	142.2 (2)	O15—N5—Sm1	55.6 (4)
O2—Sm1—O12	129.4 (2)	O2—C7—C6	124.5 (7)
O15—Sm1—O12	67.7 (2)	O2—C7—C2	118.5 (7)
O10—Sm1—O12	48.8 (2)	C6—C7—C2	117.0 (7)
O9—Sm1—O12	126.3 (2)	N4—O12—Sm1	96.7 (6)
O1—Sm1—O12	72.8 (2)	N3—O9—Sm1	100.0 (5)
O4—Sm1—O12	95.9 (2)	N3—O7—Sm1	96.0 (6)
O3—Sm1—O7	112.6 (2)	C21—O6—Zn2	125.5 (6)
O2—Sm1—O7	79.6 (2)	C21—O6—H601	116.7
O15—Sm1—O7	136.4 (2)	Zn2—O6—H601	117.8
O10—Sm1—O7	69.6 (3)	C4—C5—C6	121.3 (8)
O9—Sm1—O7	48.3 (2)	C4—C5—H5	119.4
O1—Sm1—O7	64.1 (2)	C6—C5—H5	119.4
O4—Sm1—O7	109.9 (2)	C7—C6—C5	119.0 (8)
O12—Sm1—O7	104.1 (3)	C7—C6—C8	124.2 (7)
O3—Sm1—O13	75.8 (2)	C5—C6—C8	116.8 (7)
O2—Sm1—O13	112.6 (2)	C5—C4—C3	120.6 (8)
O15—Sm1—O13	49.5 (2)	C5—C4—H4	119.7
O10—Sm1—O13	98.0 (3)	C3—C4—H4	119.7
O9—Sm1—O13	129.7 (2)	C2—C3—C4	119.1 (9)
O1—Sm1—O13	118.01 (19)	C2—C3—H3	120.4
O4—Sm1—O13	64.61 (19)	C4—C3—H3	120.4
O12—Sm1—O13	66.6 (2)	N2—C11—C10	112.2 (7)
O7—Sm1—O13	167.5 (3)	N2—C11—H11A	109.2
O3—Sm1—N5	83.26 (19)	C10—C11—H11A	109.2
O2—Sm1—N5	94.7 (2)	N2—C11—H11B	109.2
O15—Sm1—N5	24.7 (2)	C10—C11—H11B	109.2
O10—Sm1—N5	108.0 (2)	H11A—C11—H11B	107.9
O9—Sm1—N5	152.8 (2)	O1—C1—H1A	109.5
O1—Sm1—N5	95.1 (2)	O1—C1—H1B	109.5
O4—Sm1—N5	89.3 (2)	H1A—C1—H1B	109.5
O12—Sm1—N5	63.8 (2)	O1—C1—H1C	109.5
O7—Sm1—N5	158.9 (2)	H1A—C1—H1C	109.5
O13—Sm1—N5	24.83 (19)	H1B—C1—H1C	109.5
O3—Sm1—N4	147.52 (19)	C15—C16—C17	119.8 (8)
O2—Sm1—N4	143.50 (19)	C15—C16—H16	120.1
O15—Sm1—N4	91.9 (2)	C17—C16—H16	120.1
O10—Sm1—N4	24.6 (2)	N2—C12—C13	127.3 (7)
O9—Sm1—N4	102.1 (3)	N2—C12—H12	116.3
O1—Sm1—N4	79.92 (19)	C13—C12—H12	116.3
O4—Sm1—N4	85.51 (19)	C15—C14—C13	121.2 (8)
O12—Sm1—N4	24.2 (2)	C15—C14—H14	119.4
O7—Sm1—N4	87.3 (3)	C13—C14—H14	119.4
O13—Sm1—N4	81.2 (2)	C14—C15—C16	120.4 (8)
N5—Sm1—N4	85.4 (2)	C14—C15—H15	119.8
N2—Zn2—O2	168.8 (2)	C16—C15—H15	119.8
N2—Zn2—N1	99.7 (3)	N1—C8—C6	128.9 (7)
O2—Zn2—N1	90.9 (2)	N1—C8—H8	115.6
N2—Zn2—O3	90.0 (2)	C6—C8—H8	115.6
O2—Zn2—O3	79.45 (19)	N1—C9—C10	115.1 (7)
N1—Zn2—O3	170.3 (2)	N1—C9—H9A	108.5
N2—Zn2—O6	89.6 (2)	C10—C9—H9A	108.5
O2—Zn2—O6	94.4 (2)	N1—C9—H9B	108.5
N1—Zn2—O6	87.3 (2)	C10—C9—H9B	108.5
O3—Zn2—O6	92.2 (2)	H9A—C9—H9B	107.5
N2—Zn2—O5	85.5 (2)	N5—O13—Sm1	94.0 (5)
O2—Zn2—O5	90.8 (2)	O5—C20—H20A	109.5
N1—Zn2—O5	91.3 (2)	O5—C20—H20B	109.5
O3—Zn2—O5	90.1 (2)	H20A—C20—H20B	109.5
O6—Zn2—O5	174.6 (2)	O5—C20—H20C	109.5
N2—Zn2—Sm1	129.63 (19)	H20A—C20—H20C	109.5
O2—Zn2—Sm1	39.84 (13)	H20B—C20—H20C	109.5
N1—Zn2—Sm1	130.63 (19)	O11—N4—O12	122.2 (10)
O3—Zn2—Sm1	39.67 (13)	O11—N4—O10	122.8 (10)
O6—Zn2—Sm1	92.59 (17)	O12—N4—O10	114.9 (8)
O5—Zn2—Sm1	92.25 (16)	O11—N4—Sm1	178.7 (8)
C17—O4—C19	117.3 (6)	O12—N4—Sm1	59.1 (5)
C17—O4—Sm1	116.1 (4)	O10—N4—Sm1	55.9 (4)
C19—O4—Sm1	126.1 (5)	O6—C21—H21A	109.5
C2—O1—C1	116.2 (6)	O6—C21—H21B	109.5
C2—O1—Sm1	118.2 (4)	H21A—C21—H21B	109.5
C1—O1—Sm1	124.8 (5)	O6—C21—H21C	109.5
C18—O3—Zn2	124.9 (5)	H21A—C21—H21C	109.5
C18—O3—Sm1	124.8 (4)	H21B—C21—H21C	109.5
Zn2—O3—Sm1	106.2 (2)	O4—C19—H19A	109.5
C7—O2—Zn2	125.6 (5)	O4—C19—H19B	109.5
C7—O2—Sm1	125.2 (4)	H19A—C19—H19B	109.5
Zn2—O2—Sm1	106.3 (2)	O4—C19—H19C	109.5
C16—C17—O4	124.0 (7)	H19A—C19—H19C	109.5
C16—C17—C18	121.6 (7)	H19B—C19—H19C	109.5
O4—C17—C18	114.4 (6)	C23—O17—H17A	109.5
C8—N1—C9	116.7 (7)	O17—C23—H23A	109.5
C8—N1—Zn2	124.0 (6)	O17—C23—H23B	109.5
C9—N1—Zn2	119.1 (6)	H23A—C23—H23B	109.5
C20—O5—Zn2	124.8 (5)	O17—C23—H23C	109.5
C20—O5—H501	117.3	H23A—C23—H23C	109.5
Zn2—O5—H501	117.9	H23B—C23—H23C	109.5
O3—C18—C13	125.0 (7)	O16—C22—H22A	109.5
O3—C18—C17	118.1 (7)	O16—C22—H22B	109.5
C13—C18—C17	116.8 (7)	H22A—C22—H22B	109.5
N5—O15—Sm1	99.7 (5)	O16—C22—H22C	109.5
C12—N2—C11	117.8 (7)	H22A—C22—H22C	109.5
C12—N2—Zn2	125.1 (5)	H22B—C22—H22C	109.5
C11—N2—Zn2	116.8 (5)	C22—O16—H16A	109.5
			
O3—Sm1—Zn2—N2	−1.4 (3)	N1—Zn2—O5—C20	54.0 (7)
O2—Sm1—Zn2—N2	174.7 (3)	O3—Zn2—O5—C20	−116.4 (7)
O15—Sm1—Zn2—N2	−106.7 (3)	Sm1—Zn2—O5—C20	−76.7 (7)
O10—Sm1—Zn2—N2	75.6 (7)	Zn2—O3—C18—C13	−15.2 (10)
O9—Sm1—Zn2—N2	70.6 (3)	Sm1—O3—C18—C13	−169.2 (5)
O1—Sm1—Zn2—N2	−177.9 (3)	Zn2—O3—C18—C17	167.6 (5)
O4—Sm1—Zn2—N2	5.9 (3)	Sm1—O3—C18—C17	13.6 (9)
O12—Sm1—Zn2—N2	−108.8 (4)	C16—C17—C18—O3	−178.6 (7)
O7—Sm1—Zn2—N2	117.4 (3)	O4—C17—C18—O3	0.4 (9)
O13—Sm1—Zn2—N2	−59.0 (3)	C16—C17—C18—C13	4.0 (11)
N5—Sm1—Zn2—N2	−83.0 (3)	O4—C17—C18—C13	−177.0 (6)
O3—Sm1—Zn2—O2	−176.0 (3)	O3—Sm1—O15—N5	72.8 (5)
O15—Sm1—Zn2—O2	78.6 (3)	O2—Sm1—O15—N5	139.9 (5)
O10—Sm1—Zn2—O2	−99.0 (7)	O10—Sm1—O15—N5	−75.2 (6)
O9—Sm1—Zn2—O2	−104.1 (3)	O9—Sm1—O15—N5	94.6 (10)
O1—Sm1—Zn2—O2	7.5 (3)	O1—Sm1—O15—N5	−153.6 (5)
O4—Sm1—Zn2—O2	−168.8 (3)	O4—Sm1—O15—N5	11.1 (5)
O12—Sm1—Zn2—O2	76.5 (4)	O12—Sm1—O15—N5	−75.6 (5)
O7—Sm1—Zn2—O2	−57.3 (3)	O7—Sm1—O15—N5	−162.8 (5)
O13—Sm1—Zn2—O2	126.4 (3)	O13—Sm1—O15—N5	2.0 (5)
N5—Sm1—Zn2—O2	102.3 (3)	N4—Sm1—O15—N5	−74.8 (5)
O3—Sm1—Zn2—N1	179.0 (3)	O2—Zn2—N2—C12	1.2 (17)
O2—Sm1—Zn2—N1	−5.0 (3)	N1—Zn2—N2—C12	163.0 (7)
O15—Sm1—Zn2—N1	73.6 (3)	O3—Zn2—N2—C12	−17.6 (7)
O10—Sm1—Zn2—N1	−104.1 (7)	O6—Zn2—N2—C12	−109.8 (7)
O9—Sm1—Zn2—N1	−109.1 (3)	O5—Zn2—N2—C12	72.5 (7)
O1—Sm1—Zn2—N1	2.5 (3)	Sm1—Zn2—N2—C12	−16.8 (8)
O4—Sm1—Zn2—N1	−173.8 (3)	O2—Zn2—N2—C11	−172.0 (11)
O12—Sm1—Zn2—N1	71.5 (4)	N1—Zn2—N2—C11	−10.2 (6)
O7—Sm1—Zn2—N1	−62.3 (3)	O3—Zn2—N2—C11	169.2 (6)
O13—Sm1—Zn2—N1	121.3 (3)	O6—Zn2—N2—C11	77.0 (6)
N5—Sm1—Zn2—N1	97.3 (3)	O5—Zn2—N2—C11	−100.7 (6)
O2—Sm1—Zn2—O3	176.0 (3)	Sm1—Zn2—N2—C11	170.1 (5)
O15—Sm1—Zn2—O3	−105.4 (3)	O3—Sm1—O10—N4	−124.9 (5)
O10—Sm1—Zn2—O3	77.0 (7)	O2—Sm1—O10—N4	104.1 (6)
O9—Sm1—Zn2—O3	71.9 (3)	O15—Sm1—O10—N4	1.0 (7)
O1—Sm1—Zn2—O3	−176.5 (3)	O9—Sm1—O10—N4	−176.5 (6)
O4—Sm1—Zn2—O3	7.2 (3)	O1—Sm1—O10—N4	70.5 (6)
O12—Sm1—Zn2—O3	−107.5 (4)	O4—Sm1—O10—N4	−108.9 (6)
O7—Sm1—Zn2—O3	118.7 (3)	O12—Sm1—O10—N4	1.5 (5)
O13—Sm1—Zn2—O3	−57.6 (3)	O7—Sm1—O10—N4	133.7 (6)
N5—Sm1—Zn2—O3	−81.7 (3)	O13—Sm1—O10—N4	−47.5 (6)
O3—Sm1—Zn2—O6	90.3 (3)	N5—Sm1—O10—N4	−24.1 (6)
O2—Sm1—Zn2—O6	−93.7 (3)	C1—O1—C2—C3	−18.2 (12)
O15—Sm1—Zn2—O6	−15.1 (2)	Sm1—O1—C2—C3	171.2 (6)
O10—Sm1—Zn2—O6	167.3 (7)	C1—O1—C2—C7	163.1 (7)
O9—Sm1—Zn2—O6	162.2 (2)	Sm1—O1—C2—C7	−7.5 (8)
O1—Sm1—Zn2—O6	−86.2 (2)	O3—C18—C13—C14	178.9 (7)
O4—Sm1—Zn2—O6	97.5 (2)	C17—C18—C13—C14	−3.9 (10)
O12—Sm1—Zn2—O6	−17.2 (4)	O3—C18—C13—C12	−1.8 (12)
O7—Sm1—Zn2—O6	−151.0 (2)	C17—C18—C13—C12	175.5 (7)
O13—Sm1—Zn2—O6	32.6 (2)	Sm1—O15—N5—O14	177.6 (8)
N5—Sm1—Zn2—O6	8.6 (2)	Sm1—O15—N5—O13	−3.6 (8)
O3—Sm1—Zn2—O5	−87.4 (3)	O3—Sm1—N5—O13	70.5 (5)
O2—Sm1—Zn2—O5	88.6 (3)	O2—Sm1—N5—O13	137.6 (5)
O15—Sm1—Zn2—O5	167.2 (2)	O15—Sm1—N5—O13	176.4 (9)
O10—Sm1—Zn2—O5	−10.4 (7)	O10—Sm1—N5—O13	−69.2 (6)
O9—Sm1—Zn2—O5	−15.5 (2)	O9—Sm1—N5—O13	28.8 (8)
O1—Sm1—Zn2—O5	96.1 (2)	O1—Sm1—N5—O13	−158.4 (5)
O4—Sm1—Zn2—O5	−80.18 (19)	O4—Sm1—N5—O13	6.6 (5)
O12—Sm1—Zn2—O5	165.1 (4)	O12—Sm1—N5—O13	−90.4 (6)
O7—Sm1—Zn2—O5	31.3 (2)	O7—Sm1—N5—O13	−149.1 (7)
O13—Sm1—Zn2—O5	−145.1 (2)	N4—Sm1—N5—O13	−79.0 (5)
N5—Sm1—Zn2—O5	−169.1 (2)	O3—Sm1—N5—O15	−105.9 (5)
O3—Sm1—O4—C17	13.0 (5)	O2—Sm1—N5—O15	−38.7 (5)
O2—Sm1—O4—C17	0.2 (6)	O10—Sm1—N5—O15	114.5 (5)
O15—Sm1—O4—C17	91.2 (5)	O9—Sm1—N5—O15	−147.6 (5)
O10—Sm1—O4—C17	−155.4 (5)	O1—Sm1—N5—O15	25.2 (5)
O9—Sm1—O4—C17	−73.2 (5)	O4—Sm1—N5—O15	−169.8 (5)
O1—Sm1—O4—C17	−157.6 (6)	O12—Sm1—N5—O15	93.2 (5)
O12—Sm1—O4—C17	159.4 (5)	O7—Sm1—N5—O15	34.5 (10)
O7—Sm1—O4—C17	−93.2 (5)	O13—Sm1—N5—O15	−176.4 (9)
O13—Sm1—O4—C17	98.9 (5)	N4—Sm1—N5—O15	104.6 (5)
N5—Sm1—O4—C17	95.8 (5)	Zn2—O2—C7—C6	−12.6 (10)
N4—Sm1—O4—C17	−178.7 (5)	Sm1—O2—C7—C6	−170.8 (5)
O3—Sm1—O4—C19	−158.4 (7)	Zn2—O2—C7—C2	168.5 (5)
O2—Sm1—O4—C19	−171.2 (7)	Sm1—O2—C7—C2	10.3 (9)
O15—Sm1—O4—C19	−80.2 (7)	C3—C2—C7—O2	−179.6 (7)
O10—Sm1—O4—C19	33.2 (7)	O1—C2—C7—O2	−0.9 (10)
O9—Sm1—O4—C19	115.4 (7)	C3—C2—C7—C6	1.5 (12)
O1—Sm1—O4—C19	31.0 (11)	O1—C2—C7—C6	−179.8 (6)
O12—Sm1—O4—C19	−12.0 (7)	O3—Sm1—O12—N4	119.2 (6)
O7—Sm1—O4—C19	95.4 (7)	O2—Sm1—O12—N4	−135.3 (5)
O13—Sm1—O4—C19	−72.5 (7)	O15—Sm1—O12—N4	178.0 (7)
N5—Sm1—O4—C19	−75.6 (7)	O10—Sm1—O12—N4	−1.5 (5)
N4—Sm1—O4—C19	9.9 (7)	O9—Sm1—O12—N4	0.9 (7)
O3—Sm1—O1—C2	1.6 (6)	O1—Sm1—O12—N4	−103.9 (6)
O2—Sm1—O1—C2	8.9 (5)	O4—Sm1—O12—N4	65.0 (6)
O15—Sm1—O1—C2	−73.2 (5)	O7—Sm1—O12—N4	−47.3 (7)
O10—Sm1—O1—C2	168.2 (5)	O13—Sm1—O12—N4	124.0 (7)
O9—Sm1—O1—C2	92.5 (5)	N5—Sm1—O12—N4	151.2 (7)
O4—Sm1—O1—C2	170.3 (6)	O8—N3—O9—Sm1	179.0 (8)
O12—Sm1—O1—C2	−144.5 (6)	O7—N3—O9—Sm1	−3.2 (9)
O7—Sm1—O1—C2	99.8 (6)	O3—Sm1—O9—N3	140.0 (6)
O13—Sm1—O1—C2	−94.0 (5)	O2—Sm1—O9—N3	73.8 (6)
N5—Sm1—O1—C2	−83.9 (5)	O15—Sm1—O9—N3	117.5 (9)
N4—Sm1—O1—C2	−168.4 (6)	O10—Sm1—O9—N3	−71.8 (6)
O3—Sm1—O1—C1	−168.1 (6)	O1—Sm1—O9—N3	10.5 (6)
O2—Sm1—O1—C1	−160.8 (7)	O4—Sm1—O9—N3	−152.7 (6)
O15—Sm1—O1—C1	117.1 (7)	O12—Sm1—O9—N3	−73.7 (7)
O10—Sm1—O1—C1	−1.5 (7)	O7—Sm1—O9—N3	1.8 (5)
O9—Sm1—O1—C1	−77.2 (7)	O13—Sm1—O9—N3	−161.9 (5)
O4—Sm1—O1—C1	0.6 (11)	N5—Sm1—O9—N3	−177.2 (5)
O12—Sm1—O1—C1	45.8 (6)	N4—Sm1—O9—N3	−73.3 (6)
O7—Sm1—O1—C1	−69.9 (7)	O8—N3—O7—Sm1	−179.2 (9)
O13—Sm1—O1—C1	96.3 (7)	O9—N3—O7—Sm1	3.0 (9)
N5—Sm1—O1—C1	106.4 (7)	O3—Sm1—O7—N3	−46.4 (7)
N4—Sm1—O1—C1	21.9 (7)	O2—Sm1—O7—N3	−107.1 (6)
N2—Zn2—O3—C18	21.0 (6)	O15—Sm1—O7—N3	−163.1 (5)
O2—Zn2—O3—C18	−155.4 (6)	O10—Sm1—O7—N3	89.6 (6)
O6—Zn2—O3—C18	110.6 (6)	O9—Sm1—O7—N3	−1.8 (5)
O5—Zn2—O3—C18	−64.6 (5)	O1—Sm1—O7—N3	−172.8 (7)
Sm1—Zn2—O3—C18	−158.0 (7)	O4—Sm1—O7—N3	22.9 (7)
N2—Zn2—O3—Sm1	179.0 (2)	O12—Sm1—O7—N3	124.7 (6)
O2—Zn2—O3—Sm1	2.6 (2)	O13—Sm1—O7—N3	84.3 (12)
O6—Zn2—O3—Sm1	−91.5 (2)	N5—Sm1—O7—N3	176.9 (6)
O5—Zn2—O3—Sm1	93.4 (2)	N4—Sm1—O7—N3	107.1 (6)
O2—Sm1—O3—C18	155.6 (6)	N2—Zn2—O6—C21	44.4 (8)
O15—Sm1—O3—C18	−130.1 (5)	O2—Zn2—O6—C21	−125.1 (7)
O10—Sm1—O3—C18	3.4 (7)	N1—Zn2—O6—C21	144.2 (8)
O9—Sm1—O3—C18	55.3 (5)	O3—Zn2—O6—C21	−45.5 (7)
O1—Sm1—O3—C18	162.6 (5)	Sm1—Zn2—O6—C21	−85.2 (7)
O4—Sm1—O3—C18	−14.0 (5)	O2—C7—C6—C5	179.2 (7)
O12—Sm1—O3—C18	−77.9 (6)	C2—C7—C6—C5	−1.9 (11)
O7—Sm1—O3—C18	87.9 (6)	O2—C7—C6—C8	−0.2 (12)
O13—Sm1—O3—C18	−82.4 (5)	C2—C7—C6—C8	178.7 (7)
N5—Sm1—O3—C18	−106.5 (5)	C4—C5—C6—C7	1.2 (13)
N4—Sm1—O3—C18	−36.0 (7)	C4—C5—C6—C8	−179.4 (8)
O2—Sm1—O3—Zn2	−2.40 (19)	C6—C5—C4—C3	0.0 (15)
O15—Sm1—O3—Zn2	71.9 (2)	O1—C2—C3—C4	−178.8 (8)
O10—Sm1—O3—Zn2	−154.6 (3)	C7—C2—C3—C4	−0.2 (13)
O9—Sm1—O3—Zn2	−102.7 (3)	C5—C4—C3—C2	−0.5 (14)
O1—Sm1—O3—Zn2	4.6 (3)	C12—N2—C11—C10	−127.4 (9)
O4—Sm1—O3—Zn2	−172.0 (3)	Zn2—N2—C11—C10	46.3 (10)
O12—Sm1—O3—Zn2	124.1 (3)	C9—C10—C11—N2	−80.2 (10)
O7—Sm1—O3—Zn2	−70.1 (3)	O4—C17—C16—C15	179.5 (7)
O13—Sm1—O3—Zn2	119.6 (3)	C18—C17—C16—C15	−1.5 (12)
N5—Sm1—O3—Zn2	95.5 (2)	C11—N2—C12—C13	−178.4 (8)
N4—Sm1—O3—Zn2	166.0 (4)	Zn2—N2—C12—C13	8.5 (12)
N2—Zn2—O2—C7	176.7 (11)	C14—C13—C12—N2	−174.9 (8)
N1—Zn2—O2—C7	14.6 (6)	C18—C13—C12—N2	5.7 (13)
O3—Zn2—O2—C7	−164.2 (6)	C18—C13—C14—C15	1.5 (12)
O6—Zn2—O2—C7	−72.8 (6)	C12—C13—C14—C15	−177.9 (8)
O5—Zn2—O2—C7	105.9 (6)	C13—C14—C15—C16	1.1 (13)
Sm1—Zn2—O2—C7	−161.6 (7)	C17—C16—C15—C14	−1.1 (13)
N2—Zn2—O2—Sm1	−21.7 (13)	C9—N1—C8—C6	176.3 (8)
N1—Zn2—O2—Sm1	176.2 (2)	Zn2—N1—C8—C6	0.2 (12)
O3—Zn2—O2—Sm1	−2.6 (2)	C7—C6—C8—N1	7.1 (14)
O6—Zn2—O2—Sm1	88.8 (2)	C5—C6—C8—N1	−172.3 (9)
O5—Zn2—O2—Sm1	−92.5 (2)	C8—N1—C9—C10	160.4 (8)
O3—Sm1—O2—C7	164.1 (6)	Zn2—N1—C9—C10	−23.3 (11)
O15—Sm1—O2—C7	67.7 (5)	C11—C10—C9—N1	66.1 (11)
O10—Sm1—O2—C7	−48.1 (7)	O14—N5—O13—Sm1	−177.8 (8)
O9—Sm1—O2—C7	−122.4 (5)	O15—N5—O13—Sm1	3.4 (8)
O1—Sm1—O2—C7	−10.0 (5)	O3—Sm1—O13—N5	−105.0 (5)
O4—Sm1—O2—C7	176.5 (5)	O2—Sm1—O13—N5	−46.7 (6)
O12—Sm1—O2—C7	23.7 (6)	O15—Sm1—O13—N5	−2.0 (5)
O7—Sm1—O2—C7	−76.2 (5)	O10—Sm1—O13—N5	116.2 (5)
O13—Sm1—O2—C7	101.2 (5)	O9—Sm1—O13—N5	−163.4 (5)
N5—Sm1—O2—C7	83.3 (5)	O1—Sm1—O13—N5	24.5 (6)
N4—Sm1—O2—C7	−5.4 (7)	O4—Sm1—O13—N5	−172.7 (6)
O3—Sm1—O2—Zn2	2.41 (19)	O12—Sm1—O13—N5	78.0 (5)
O15—Sm1—O2—Zn2	−94.0 (3)	O7—Sm1—O13—N5	121.1 (10)
O10—Sm1—O2—Zn2	150.2 (3)	N4—Sm1—O13—N5	98.1 (5)
O9—Sm1—O2—Zn2	75.9 (3)	Sm1—O12—N4—O11	179.7 (8)
O1—Sm1—O2—Zn2	−171.7 (3)	Sm1—O12—N4—O10	2.5 (9)
O4—Sm1—O2—Zn2	14.8 (3)	Sm1—O10—N4—O11	−179.8 (8)
O12—Sm1—O2—Zn2	−138.0 (3)	Sm1—O10—N4—O12	−2.6 (9)
O7—Sm1—O2—Zn2	122.2 (3)	O3—Sm1—N4—O12	−95.5 (7)
O13—Sm1—O2—Zn2	−60.5 (3)	O2—Sm1—N4—O12	66.1 (8)
N5—Sm1—O2—Zn2	−78.4 (2)	O15—Sm1—N4—O12	−1.9 (6)
N4—Sm1—O2—Zn2	−167.1 (3)	O10—Sm1—N4—O12	177.3 (10)
C19—O4—C17—C16	−20.7 (11)	O9—Sm1—N4—O12	−179.2 (6)
Sm1—O4—C17—C16	167.1 (6)	O1—Sm1—N4—O12	70.3 (6)
C19—O4—C17—C18	160.3 (7)	O4—Sm1—N4—O12	−115.3 (6)
Sm1—O4—C17—C18	−11.9 (8)	O7—Sm1—N4—O12	134.5 (6)
N2—Zn2—N1—C8	174.9 (7)	O13—Sm1—N4—O12	−50.3 (6)
O2—Zn2—N1—C8	−8.6 (7)	N5—Sm1—N4—O12	−25.7 (6)
O6—Zn2—N1—C8	85.8 (7)	O3—Sm1—N4—O10	87.2 (7)
O5—Zn2—N1—C8	−99.4 (7)	O2—Sm1—N4—O10	−111.2 (6)
Sm1—Zn2—N1—C8	−5.3 (8)	O15—Sm1—N4—O10	−179.1 (6)
N2—Zn2—N1—C9	−1.0 (7)	O9—Sm1—N4—O10	3.5 (6)
O2—Zn2—N1—C9	175.5 (6)	O1—Sm1—N4—O10	−106.9 (6)
O6—Zn2—N1—C9	−90.2 (6)	O4—Sm1—N4—O10	67.4 (6)
O5—Zn2—N1—C9	84.7 (6)	O12—Sm1—N4—O10	−177.3 (10)
Sm1—Zn2—N1—C9	178.7 (5)	O7—Sm1—N4—O10	−42.8 (6)
N2—Zn2—O5—C20	153.7 (7)	O13—Sm1—N4—O10	132.4 (6)
O2—Zn2—O5—C20	−36.9 (7)	N5—Sm1—N4—O10	157.0 (6)

### Hydrogen-bond geometry (Å, °)

**Table:**

*D*—H···*A*	*D*—H	H···*A*	*D*···*A*	*D*—H···*A*
O5—H501···O8^i^	0.82	2.38	3.107 (11)	148
O6—H601···O17^ii^	0.82	1.99	2.654 (10)	138
O17—H17A···O16	0.82	1.88	2.688 (13)	167

Symmetry codes: (i) −*x*+1, −*y*+1, −*z*; (ii) −*x*+2, *y*+1/2, −*z*+1/2.
